# A proteomic study of *Corynebacterium glutamicum *AAA+ protease FtsH

**DOI:** 10.1186/1471-2180-7-6

**Published:** 2007-01-25

**Authors:** Alja Lüdke, Reinhard Krämer, Andreas Burkovski, Daniela Schluesener, Ansgar Poetsch

**Affiliations:** 1 Institut für Biochemie, Universität zu Köln, Zülpicher Str. 47, 50674 Köln, Germany; 2 Lehrstuhl für Mikrobiologie, Friedrich-Alexander-Universität Erlangen-Nürnberg, Staudtstr. 5, 91058 Erlangen, Germany; 3 Lehrstuhl für Biochemie der Pflanzen, Ruhr-Universität Bochum, Universitätsstr. 150, 44801 Bochum, Germany

## Abstract

**Background:**

The influence of the membrane-bound AAA+ protease FtsH on membrane and cytoplasmic proteins of *Corynebacterium glutamicum *was investigated in this study. For the analysis of the membrane fraction, anion exchange chromatography was combined with SDS-PAGE, while the cytoplasmic protein fraction was studied by conventional two-dimensional gel electrophoresis.

**Results:**

In contrast to the situation in other bacteria, deletion of *C. glutamicum ftsH *has no significant effect on growth in standard minimal medium or response to heat or osmotic stress. On the proteome level, deletion of the *ftsH *gene resulted in a strong increase of ten cytoplasmic and membrane proteins, namely biotin carboxylase/biotin carboxyl carrier protein (*accBC*), glyceraldehyde-3-phosphate dehydrogenase (*gap*), homocysteine methyltransferase (*metE*), malate synthase (*aceB*), isocitrate lyase (*aceA*), a conserved hypothetical protein (NCgl1985), succinate dehydrogenase A (*sdhA*), succinate dehydrogenase B (*sdhB*), succinate dehydrogenase CD (*sdhCD*), and glutamate binding protein (*gluB*), while 38 cytoplasmic and membrane-associated proteins showed a decreased abundance. The decreasing amount of succinate dehydrogenase A (*sdhA*) in the cytoplasmic fraction of the *ftsH *mutant compared to the wild type and its increasing abundance in the membrane fraction indicates that FtsH might be involved in the cleavage of a membrane anchor of this membrane-associated protein and by this changes its localization.

**Conclusion:**

The data obtained hint to an involvement of *C. glutamicum *FtsH protease mainly in regulation of energy and carbon metabolism, while the protease is not involved in stress response, as found in other bacteria.

## Background

*Corynebacterium glutamicum*, is a Gram-positive soil bacterium, which is used for the industrial production of different amino acids, mainly L-glutamate and L-lysine, and of nucleotides [1,2]. As a member of the Corynebacterinae, *C. glutamicum *is closely related to other mycolic acid-containing bacteria, *e. g*. to the amino acids producer *Corynebacterium efficiens *and to important pathogens such as *Corynebacterium diphtheriae*, *Mycobacterium tuberculosis *and *Mycobacterium leprae *[3]. Due to the enormous industrial importance of *C. glutamicum*, this bacterium is very well investigated. Its genome was sequenced and published independently by different industry-supported groups recently [4,5] and different global analyses techniques are available including transcriptome [6], metabolome [7], flux [8] and proteome analyses [9].

We are interested in nitrogen metabolism and nitrogen control in *C. glutamicum *(for review, see [10-12]) and recently identified proteolysis as a new regulatory mechanism in nitrogen regulation [13]. Different proteases, namely ClpXP and ClpCP [14] as well as FtsH are involved in the degradation of nitrogen signal transduction protein GlnK [13]. The identified enzymes are members of the AAA+ protease family. These proteases and protein disassembly machines are found in all kingdoms of life and often exhibit crucial regulatory functions (for recent reviews, see [15,16]).

In *C. glutamicum*, an effect of FtsH on the degradation of nitrogen signal control protein GlnK was reported [13]. The deletion of the *ftsH *gene is very well tolerated by *C. glutamicum *cells and obvious detrimental effects of an *ftsH *deletion could not be observed. Since we were interested in the function of this protease, we initiated a proteomic study and investigated the influence of an *ftsH *deletion on membrane and cytoplasmic protein profiles.

## Results

### Influence of FtsH on growth of *C. glutamicum *strains

**Figure 1:**
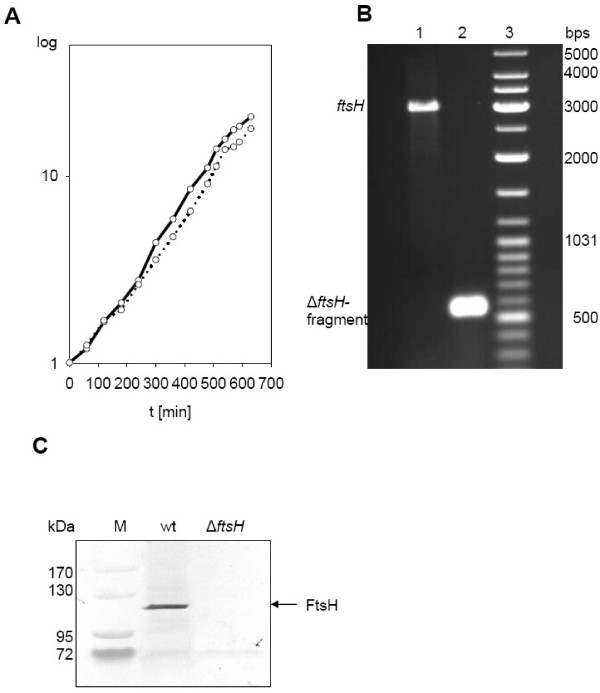
(A) Growth of the wild type strain ATCC 13032 (black line) and the deletion mutant ATCC 13032 Δ*ftsH *(dotted line). Doubling times of the wild type and the mutant strain were very similar (2 h 18 min versus 2 h 38 min). (B) Control of *ftsH *deletion by PCR. Primers were designed to anneal approx. 214 bps up and down stream of *ftsH *gene (2562 bps). The PCR product comprised 2990 bps in the wild type strain (lane 1) and 530 bps in the deletion mutant (lane 2), lane 3 contains marker DNA. (C) Control of *ftsH *deletion by Western blotting. 25 μg of membrane protein of the wild type and Δ*ftsH *were applied per lane. No signal was obtained with cytoplasmic proteins (data not shown).

Mutations of *ftsH *were described in different bacteria. The effect of these mutations are remarkably species-specific and range from drastic growth impairment in *Escherichia coli *[17] to effects on sporulation, development and stress response in *Bacillus subtilis *[18,19] and *Caulobacter crescentus *[20]. When growth of *C. glutamicum *wild type strains ATCC 13032 and strain Δ*ftsH *was analyzed, only a minor effect of the *ftsH *deletion was observed (Fig. 1A). Doubling times of wild type and mutant strain were very similar in standard minimal medium (2 h 18 min versus 2 h 38 min). Next, the influence of increased temperature on growth was tested. Neither growth at increased temperature (37°C instead of 30°C) had a significant detrimental effect nor exposure of cells grown at 30°C to a sudden heat shock of 37 and 39°C, respectively (data not shown). Also when the *ftsH *mutant was exposed to osmotic stress applied either by growth in medium with increased osmolarity or as sudden osmotic shock due to sodium chloride addition, no significant growth defect compared to the wild type was detectable (S. Morbach and U. Meyer, personal communication). Obviously, FtsH plays a less crucial role in *C. glutamicum *compared to other bacteria. To identify FtsH targets in the cell, proteome studies were carried out.

### Differences in the membrane proteome of wild type and *ftsH *deletion strain

FtsH is a membrane-bound AAA+ protease and therefore we started our investigations with an analysis of membrane proteins. While the separation of *C. glutamicum *membrane proteins by 2-D PAGE is restricted to those with two or less transmembrane helices [21,22], recently, a technique was established to separate highly hydrophobic proteins of the membrane fraction by anion exchange chromatography and 1-D SDS-PAGE [23]. This technique was applied for the comparison of membrane proteins from wild type and corresponding *ftsH *deletion strain.

**Figure 2:**
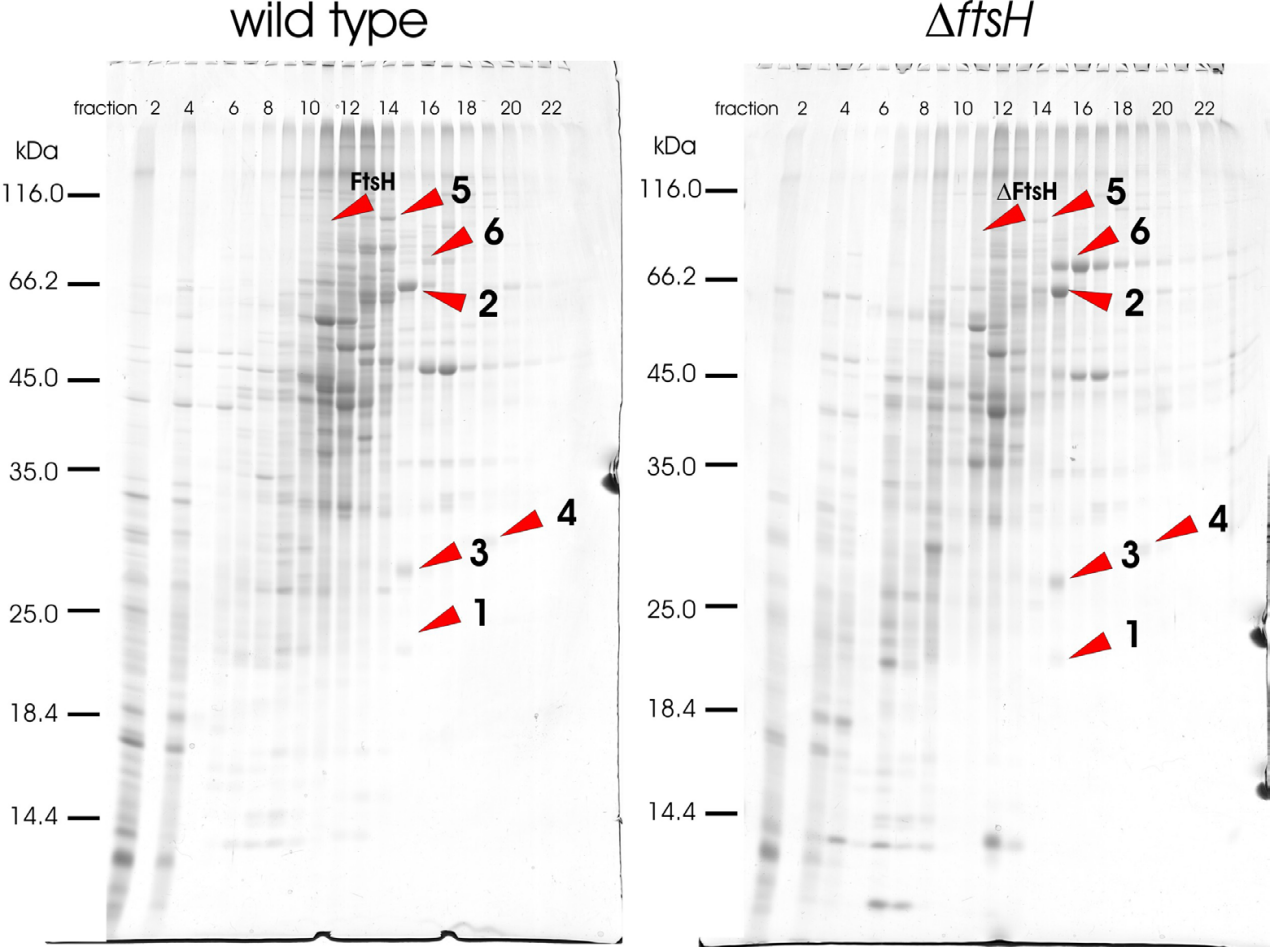
Coomassie-stained 1-D gels after ion exchange chromatography of wild type and *ftsH *deletion mutant membrane fraction. Protein spots which appear to be regulated are marked by red arrow heads and are listed in Table 1.

The FtsH protease was identified for the wild type in a faint gel band in all three biological replicates, while this band was absent in the deletion strain (Figure 2). Compared to the wild type, five different proteins showed an increased abundance in the *ftsH *mutant strain (Table 1), namely all subunits of the succinate dehydrogenase complex (*sdhA, sdhB *and *sdhCD*), glutamate binding protein (*gluB*) and homocysteine methyltransferase (*metE*). Upregulation of protein concentration did not exceed a factor of five. While the succinate dehydrogenase complex could be a direct substrate of FtsH, GluB is a lipid-anchored glutamate binding protein [24], which is located at the outer face of the cytoplasmic membrane. Therefore, GluB must be an indirect target or processed by FtsH before secretion to the external site of the cell. *C. glutamicum *contains two homocysteine methyltransferares (*metE *and *metH*) catalyzing the final reaction of methionine synthesis, yet only *metH *requires vitamin B_12 _(cobalamin) as a cofactor [25]. Upregulation of *metE *could indicate that more of this enzyme is required for methionine synthesis, maybe due to reduced import of cobalamin in the *ftsH *deletion mutant, though further experiments are needed to verify this hypothesis. Interestingly, the *ftsH *deletion also influenced the abundance of ClpC, the ATPase component of the ClpCP protease complex. This protein was down-regulated in the mutant compared to the wild type by a factor of 0.4. However, the significance of this putative cross-talk between AAA+ proteases in *C. glutamicum *needs further investigation.

Additionally, a role of FtsH in the response to nitrogen starvation and to improving nitrogen conditions after a starvation period was tested. Compared to the wild type, NADH dehydrogenase (*ndh*), a putative integral membrane protein (*cg0952*) and the ATPase component of an ABC-type sugar transport system (*msiK*) were down-regulated by a factor of 0.5 (data not shown).

### Comparison of cytoplasmic protein profiles

**Figure 3:**
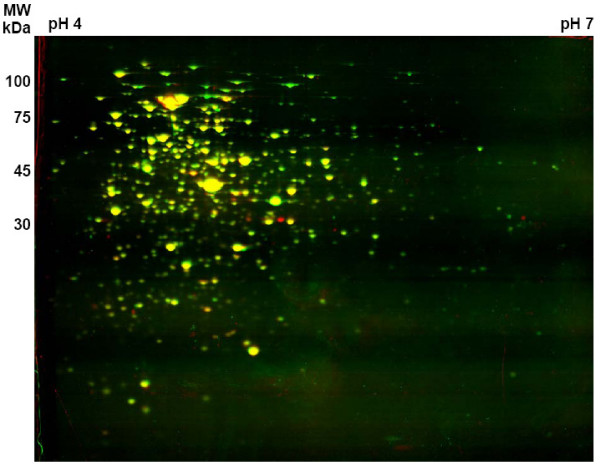
Comparison of cytoplasmic proteins of wild type strain ATCC 13032 and strain Δ*ftsH*. Overlay of 2-D gels and false colour presentation: wild type proteins were stained in green, proteins of the deletion mutant in red. Spots present in both protein profiles appear in yellow. Molecular mass and pH range are indicated.

**Figure 4:**
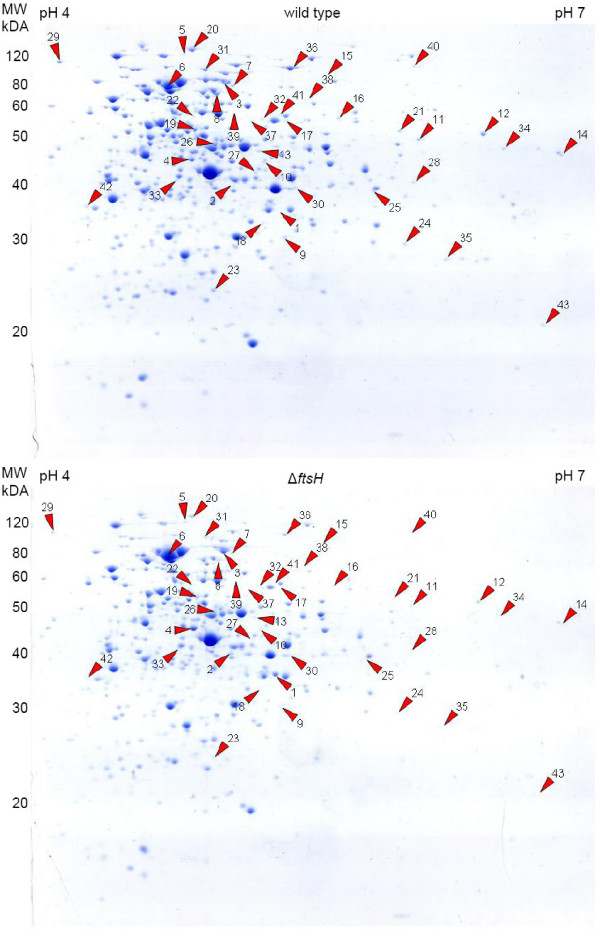
Coomassie-stained 2-D gels of wild type ATCC 13032 and corresponding *ftsH *deletion mutant. Protein spots which appear to be regulated are marked by red arrow heads and are listed in Table 2. Molecular mass and pH range are indicated.

In addition to the membrane proteome also the cytoplasmic protein fraction of wild type and *ftsH *deletion was analyzed by two-dimensional gel electrophoresis (2-D PAGE, Figure 3). By this approach, six proteins were found in increasing amounts, namely the biotin carboxylase/biotin carrier protein (*accBC*), glyceraldehyde-3-phosphate dehydrogenase (*gap*), malate synthase (*aceB*), isocitrate lyase (*aceA*), a conserved hypothetical protein (NCgl1985) and homocysteine methyltransferase (*metE*), which was also identified as an upregulated protein in the membrane fraction. AccBC was upregulated more than 50-fold, while GAP-DH was upregulated by a factor of four. 37 different protein spots showed a decreased abundance in the mutant. Almost one third of the proteins identified (presented in Table 2) is clearly involved in carbon and energy metabolism. These include the maltooligosyl trehalose synthase (*treY*), the 1,4-alpha-glucan branching enzyme (*glgB*), fumarate hydratase (*fum*), a putative L-lactate dehydrogenase (*lldA*), glyceraldehyde-3-phosphate dehydrogenase (*gap*), phosphoenolpyruvate carboxylase (*ppc*), pyruvate dehydrogenase E1 component (*aceE*), an acyl-CoA synthetase (*fadD4*), succinate dehydrogenase A (*sdhA*) and transaldolase (*tal*). Interestingly, GAPDH is present in two spots, which differ in their approximate pI, an upregulated one (see above) spot number 2 in Figure 4 and a downregulated spot (number 30), indicating a posttranslational modification of the protein. Furthermore in the *ftsH *deletion strain succinate dehydrogenase A (*sdhA*) is less present in the cytoplasm but enriched in the membrane fraction. This indicates that FtsH is involved, either directly or indirectly, in the release of this succinate dehydrogenase subunit from the membrane into the cytoplasm. Since FtsH lacks a robust unfolding activity, a cleavage of SdhA and release of the protein from the complex by FtsH is rather unlikely. Other identified proteins with decreased abundance were parts of amino acid metabolism such as glutamine synthetase (*glnA*), aspartate-ammonium-lyase (*aspA*), succinyl-diaminopimelate desuccinylase (*dapE*), and dihydroxy-acid dehydratase (*ilvD*) (Fig. 4). As in the case of the membrane proteome, the influence of FtsH on the cytoplasmic protein profile in dependence of the nitrogen status of the cell was analyzed. For unknown reasons, 2-D gels of proteins isolated from nitrogen-starved Δ*ftsH *cells revealed in contrast to the wild type (see also [26]) reproducibly strong horizontal streaking. The reason for this FtsH-specific effect, which made comparisons impossible, is unknown and could not be prevented by alternative gel loading techniques such as cup loading (C. Lück, personal communication).

## Discussion

Data which hint to an involvement of FtsH in GlnK signal transduction protein degradation [13] prompted us to investigate the influence of this AAA+ protease on membrane and cytoplasmic protein profiles in *C. glutamicum*. Using a combination of anion exchange chromatography and SDS-PAGE for membrane protein analysis and 2-D PAGE for cytoplasmic proteins, we were able to show that FtsH regulates only a few proteins under the growth conditions tested. However, since the applied method only covers about 10% of the *C. glutamicum *membrane proteome [23], some FtsH targets may have been missed due to technical limitations. For example, the FtsH target GlnK is degraded depending on FtsH but proteolysis is also influenced by ClpCP and ClpXP [13]. In contrast to the situation in *E. coli *(for recent review, see [27]), we found that deletion of the *ftsH *gene is tolerated by *C. glutamicum *cells very well, although this gene is conserved in all other corynebacterial genome sequences published so far, i. e. in the *C. diphtheriae *[28], *C. efficiens *[29] and *Corynebacterium jeikeium *[30] genome, and although no obvious paralog of the *ftsH *gene is encoded in the *C. glutamicum *genome. Obvious detrimental effects of an *ftsH *deletion were not observed. In this respect the *C. glutamicum *results resemble the situation in *B. subtilis *and *C. crescentus*. Also for these organisms, a less severe effect of *ftsH *mutation compared to an *E. coli *mutant was shown. In *B. subtilis*, FtsH is involved in sporulation, stress adaptation and protein secretion [18,19], and the effect of its deletion on the cytosolic proteome has been studied [31]. FtsH deletion resulted in increased levels of an arginase, a protein similar to a quinone oxidoreductase, and penicillin binding protein, but for the latter direct proteolytic action could be excluded and for the other two proteins it was not verified. FtsH of *M. tuberculosis*, which is phylogenetically closely related to *C. glutamicum*, was heterologously expressed in *E. coli*, and proteolytic activity against the known *E. coli *substrates heat shock transcription factor σ^32 ^protein, protein translocation subunit SecY, and bacteriophage λCII repressor protein was observed [32]. For *M. tuberculosis *no experimental verification exists if SecY is indeed a target of FtsH, and our data for *C. glutamicum*does not support this hypothesis, but it does not completely rule this out, too. For *C. crescentus *an involvement of FtsH in development, stress response and heat shock control was shown [20]. The *ftsH *gene is expressed transiently after temperature upshift and in stationary phase in this organism, while during normal growth conditions FtsH is dispensable. In *C. crescentus *a mutation of *ftsH *influences chaperones, DnaK is derepressed under normal temperature compared to the wild type, while an influence on GroEL abundance was not observed. In contrast, the *ftsH *deletion in *C. glutamicum *had no influence on DnaK and even less GroEL was observed compared to the wild type. Further differences besides chaperone activation are sporulation and cell cycle proteins, processes which are absent in *C. glutamicum*. The majority of proteins identified to be differentially synthesized in dependence of FtsH *C. glutamicum *seem to be involved in carbon and energy metabolism.

## Conclusion

The data obtained in this study, indicate that *C. glutamicum *AAA+ metalloprotease FtsH is not involved in the cellular response to heat or osmotic stress as shown in other bacteria. An astonishingly small amount of membrane and cytoplasmic proteins is affected by an *ftsH *deletion. From these data an involvement of FtsH in regulation of energy and carbon metabolism as well as in amino acid biosynthesis is indicated.

## Methods

### Strains and growth conditions

*C. glutamicum *type strain ATCC 13032 [33] and *ftsH *deletion mutant [13] were routinely grown on a rotary shaker at 30°C. A fresh *C. glutamicum *culture in BHI medium was used to inoculate minimal medium (per litre 42 g MOPS, 20 g (NH_4_)_2_SO_4_, 5 g urea, 0.5 g K_2_HPO_4 _× 3 H_2_O, 0.5 g KH_2_PO_4_, 0.25 g MgSO_4 _× 7 H_2_O, 0.01 g CaCl_2_, 50 g glucose, 0.2 mg biotin, 10 mg FeSO_4_, 10 mg MnSO_4_, 1 mg ZnSO_4_, 0.2 mg CuSO_4_, 0.02 mg NiCl_2 _× 6 H_2_O, 0.09 mg H_3_BO_3_, 0.06 mg CoCl_2 _× 6 H_2_O, 0.009 mg NaMoO_4 _× 2 H_2_O; pH adjusted to pH 7.0 using NaOH; [34]) for overnight growth. This culture, with an overnight OD_600 _of approximately 25 to 30, was used to inoculate fresh minimal medium to an OD_600 _of approximately 1, and cells were grown for 4 to 6 hours until the exponential growth phase was reached (OD_600 _approximately 4–5). To induce nitrogen starvation, cells were harvested by centrifugation and the pellet was suspended in and transferred to pre-warmed minimal medium without nitrogen source. The nitrogen-deprived cells were incubated at 30°C under aeration.

### Polymerase chain reaction

To verify the deletion of the *ftsH *gene, PCR experiments were carried out. Primers were designed to anneal approx. 214 bps up and down stream of *ftsH *gene (2562 bps) (ftsH+200up fw: 5'-GTG GGC TAC GGA CTT GAT TTC G-3'; ftsH+200down rv: 5'-GAA CCA ACT CTT CAT GGC CCT C-3'). Chromosomal DNA prepared by phenol-chloroform extraction was used as template. PCR was performed using *Taq *polymerase and the following program: 95°C 5 min; 30 cycles (95°C 30 s; 64°C 30 s; 72°C 3 min) followed by 72°C 10 min and cooling down to 4°C. PCR products were analyzed by agarose gel electrophoresis [35].

### SDS-PAGE and Western blotting

To demonstrate deletion of *ftsH *on protein level, cells were disrupted band fractionated as described below or 2-D PAGE. Cytoplasmic proteins and membrane fraction of the wild type ATCC13032 and the deletion strain were separated by Tricine-buffered 9.5% SDS gels as described [36]. After SDS-PAGE proteins were transferred onto a polyvinylidene difluoride membrane (PVDF, Immobilon-P, pore size 0.45 μm, Millipore, Bedford, MA, USA) by semi-dry electroblotting. Immunodetection of FtsH was performed with antibodies against peptide fragments of *E. coli *FtsH, produced in rabbit. Antibody binding was visualised by using appropriate anti-antibodies coupled to alkaline phosphatase (Sigma-Aldrich, Traufkirchen, Germany) and the BCIP/NBT alkaline phosphatase substrate (Sigma-Aldrich, Traufkirchen, Germany).

### Membrane proteomics

For analysis of membrane proteins, a combination of anion exchange chromatography and SDS-PAGE was applied as described previously [23]. For this method, cells were disrupted by French Press treatment; the membrane fraction was separated from cell debris and cytoplasm by differential (ultra)centrifugation and washed with 2.5 M NaBr to remove membrane-associated proteins. Membrane proteins were subsequently solubilised in buffer containing 2% ASB-14 and separated by anion exchange chromatography. After TCA precipitation and SDS-PAGE, gels were scanned and analyzed using the LabScan software package (Amersham Biosciences, Freiburg, Germany). The scanner was calibrated with a greyscale marker (Kodak) and the same settings applied for all gels. Scanning was carried out at 300 dpi and 8 bit greyscale. Gel bands were quantified relative to each other by densitometry using the software scion image (version beta 4.0.2; Scion Corporation). Proteins from three independent experiments (biological replicates) were regarded as regulated if a p-value < 0.1 was calculated for a Student's t-test (paired, two-tailed).

### 2-D PAGE, staining and protein analysis

For 2-D PAGE analyses *C. glutamicum *cells were disrupted using glass beads and a Q-BIOgene FastPrep FP120 instrument (Q-BIOgene, Heidelberg, Germany) by lyzing the cells four times for 30 sec and 6.5 m sec^-1 ^in the presence of the proteinase inhibitor Complete as recommended by the supplier (Roche, Basel, Switzerland). Proteins were separated by ultracentrifugation in cytoplasmic and membrane-associated protein fractions [37,21]. For classical 2-D PAGE, only the cytoplasmic proteins were analyzed further. Protein concentrations were determined according to Dulley and Grieve [38]. For isoelectric focusing (IEF) 24 cm pre-cast IPG strips pI 4–7 and an IPGphor IEF unit (Amersham Biosiences, Freiburg, Germany) were used as described [39]. 100 μg and 200 μg of protein were loaded by rehydration for 24 h in a sample buffer containing 6 M urea, 2 M thiourea, 4% CHAPS, 0.5% Pharmalyte (3–10) and 0.4% DTT. The isoelectric focusing was performed for 48 000 Vh. The run for the second dimension was carried out using 12.5% polyacrylamide gels and an Ettan Dalt II system (Amersham Biosiences, Freiburg, Germany). After electrophoresis 2-D gels were stained with Coomassie brilliant Blue [35]. The Coomassie-stained gels were aligned using the Delta2D software, version 3.3 (Decodon, Greifswald, Germany). All samples were separated at least twice by 2-D PAGE to minimize irregularities (technical replicates). To validate the results, each comparison of interest was performed using samples from at least three independent experiments (biological replicates). The Delta2D software (version 3.3) was also used for spot quantification. Proteins were regarded as regulated if (i) the corresponding ratios referring to the relative volumes of the spots changed more than two-fold and if (ii) this regulation pattern was found in all biological and technical replicates. All other proteins were classified as "not regulated". Pearson coefficients for wild type gels were higher than 0.9962, for *ftsH *gels 0.9929, and for the comparisons of wild type and *ftsH *mutant 0.9510.

### Protein identification

Protein spots or bands with significantly altered abundance in the *ftsH *mutant compared to the wild type were analyzed by trypric in-gel digest and MALDI-ToF-MS as described earlier [26].

## Authors' contributions

AL carried out growth experiments and 2-D PAGE, RK supported the project by discussions, AB supervised the experiments and was responsible for the draft of the manuscript, DS analyzed the membrane proteome and was supervised by AP. AP additionally wrote the final version of the manuscript. All authors read and approved the final manuscript.

## Figures and Tables

**Table 1:** Protein pattern of the membrane fraction of the wild type ATCC13032 and *ftsH *deletion mutant.

Spot #	NCgl #	Protein (Gene)	Δ*ftsH*/wildtype ratio	S.D.	p-value
1	0359	succinate dehydrogenase CD (*sdhCD*)	1.6	0.4	0.0804
2	0360	succinate dehydrogenase A (*sdhA*)	4.5	0.2	0.0003
3	0361	succinate dehydrogenase B (*sdhB*)	2.4	0.9	0.0916
4	1876	glutamate binding protein (*gluB*)	2.2	0.7	0.0734
5	2585	ATP-dependent protease (*clpC*)	0.4	0.03	0.0045
6	1094	homocysteine methyltransferase (*metE*)	4.9	1.4	0.0147
FtsH	2603	cell division protein	-		

**Table 2:** Cytoplasmic protein pattern of wild type strain ATCC13032 and *ftsH *deletion mutant. The listed proteins differ in their abundance of a factor of at least two.

Spot #	NCgl #	Protein (Gene)	Δ*ftsH*/wild type ratio	MW kDa	pI
1	0670	biotin carboxylase/biotin carboxyl carrier protein (*accBC*)	53.48	63.5	5.02
2	1526	glyceraldehyde-3-phosphate dehydrogenase (*gap*)	4.24	36.2	5.16
3	2247	malate synthase (*aceB*)	2.95	82.5	5.0
4	2248	isocitrate lyase (*aceA*)	2.65	47.2	4.92
5	1985	conserved hypothetical protein	2.31	122.8	4.85
6	1094	homocysteine methyltransferase (*metE*)	2.16	81.3	4.78
7	2037	maltooligosyl trehalose synthase (*treY*)	0.48	90.5	5.03
8	1177	1,4-alpha-glucan branching enzyme (*glgB*)	0.48	82.6	4.99
9	1023	putative nicotinate-nucleotide pyrophosphorylase	0.47	29.4	5.22
10	2431	nicotinic acid phosphoribosyltransferase	0.47	48.0	5.22
11	0187	L-gulonolactone oxidase	0.47	53.0	5.68
12	0578	inositol-monophosphate dehydrogenase (*guaB2*)	0.47	53.4	5.99
13	0094	AMP nucleosidase (*amn*)	0.46	53.7	5.23
14	0358	transcriptional regulator, MerR family (*ramB*)	0.46	53.9	6.29
15	0704	putative DNA helicase	0.46	84.1	5.35
16	2718	sulfite reductase (hemoprotein) (*cysI*)	0.43	63.0	5.53
17	0251	catalase (*katA*)	0.42	58.7	5.18
18	0200	quinone oxidoreductase	0.41	33.2	4.99
19	2133	glutamine synthetase (*glnA*)	0.41	53.3	4.90
20	0471	DNA-directed RNA polymerase beta chain (*rpoB*)	0.41	128.8	4.86
21	1446	aspartate ammonia-lyase (aspartase) (*aspA*)	0.4	57.6	5.69
22	1440	ATPases of the AAA+ class	0.4	58	4.91
23	1835	polyphosphate glucokinase (*ppgK*)	0.4	26.7	4.97
24	0371	probable formyltetrahydrofolate deformylase protein (*purU*)	0.39	34.3	5.68
25	2986	N-acetymuramyl-L-alanine amidase (*cwlM*)	0.38	44.5	5.63
26	0967	fumarate hydratase (*fum*)	0.37	49.8	5.06
27	1442	aspartyl aminopeptidase (*pepC*)	0.36	44.9	5.10
28	2817	putative L-lactate dehydrogenase (*lldA*)	0.34	45.7	5.72
29	2126	dihydrolipoamide succinyltransferase (*sucB*)	0.34	70.9	4.26
30	1526	glyceraldehyde-3-phosphate dehydrogenase (*gap*)	0.34	36.0	5.16
31	1523	phosphoenolpyruvate carboxylase (*ppc*)	0.33	103.2	4.92
32	0251	catalase (*katA*)	0.29	58.7	5.18
33	1064	succinyl-diaminopimelate desuccinylase (*dapE*)	0.29	40.0	4.84
34	2586	inositol-monophosphate dehydrogenase (*guaB1*)	0.28	50.8	6.39
35	2487	GCN5-related N-acetyltransferase	0.27	32.1	5.86
36	2167	pyruvate dehydrogenase E1 component (*aceE*)	0.27	102.8	5.26
37	1151	acyl-CoA synthetase (*fadD4*)	0.26	63.7	5.08
38	0360	succinate dehydrogenase A (*sdhA*)	0.25	74.7	5.37
39	0570	predicted carbohydrate kinase	0.19	60.0	5.08
40	0707	superfamily II DNA/RNA helicase, SNF2 family	0.16	106.9	5.65
41	1219	dihydroxy-acid dehydratase (*ilvD*)	0.16	64.2	5.18
42	1513	transaldolase (*tal*)	0.15	38.3	4.47
43	2602	GTP cyclohydrolase (*folE*)	0.08	22.0	6.08
